# Preceramic Polymers for Additive Manufacturing of Silicate Ceramics

**DOI:** 10.3390/polym15224360

**Published:** 2023-11-08

**Authors:** Fateme Sarraf, Sergey V. Churakov, Frank Clemens

**Affiliations:** 1 Empa-Swiss Federal Laboratories for Materials Science and Technology, Ueberlandstrasse 129, CH-8600 Dübendorf, Switzerland; 2 Institute of Geological Sciences, University of Bern, Hochschulstrasse 6, CH-3012 Bern, Switzerland; sergey.churakov@unibe.ch; 3 Paul Scherrer Institute, Forschungsstrasse 111, CH-5232 Villigen, Switzerland

**Keywords:** preceramic polymer, polysilsesquioxane, silicate, additive manufacturing, 3D print

## Abstract

The utilization of preceramic polymers (PCPs) to produce both oxide and non-oxide ceramics has caught significant interest, owing to their exceptional characteristics. Diverse types of polymer-derived ceramics (PDCs) synthesized by using various PCPs have demonstrated remarkable characteristics such as exceptional thermal stability, resistance to corrosion and oxidation at elevated temperatures, biocompatibility, and notable dielectric properties, among others. The application of additive manufacturing techniques to produce PDCs opens up new opportunities for manufacturing complex and unconventional ceramic structures with complex designs that might be challenging or impossible to achieve using traditional manufacturing methods. This is particularly advantageous in industries like aerospace, automotive, and electronics. In this review, various categories of preceramic polymers employed in the synthesis of polymer-derived ceramics are discussed, with a particular focus on the utilization of polysiloxane and polysilsesquioxanes to generate silicate ceramics. Further, diverse additive manufacturing techniques adopted for the fabrication of polymer-derived silicate ceramics are described.

## 1. Preceramic Polymers

The idea of using molecular precursors to produce ceramic structures was first introduced by Ainger and Herbert in 1960 [1]. Pyrolysis of organosilicon polymers to produce ceramic materials for use in high-temperature applications was presented by Verbeek in the early 1970s. The process was specifically designed to make Si_3_N_4_/SiC ceramic fibers [2]. The research study of Yajima et al. in 1975 on the synthesis of SiC-based fibers from polycarbosilane marked a major advancement in the area of polymer pyrolysis for the production of polymer-derived ceramics (PDCs) [3]. Since then, preceramic polymers (PCPs), more specifically organosilicon polymers, have been widely acknowledged as an effective method to create advanced ceramics. It is worthwhile to mention that different terms have been used for organosilicon polymers [4,5] such as Si-based polymers [6,7], Si-based preceramic polymers [8,9], and silicone resins [10,11].

### 1.1. Si-Based Preceramic Polymers

**Figure 1:**
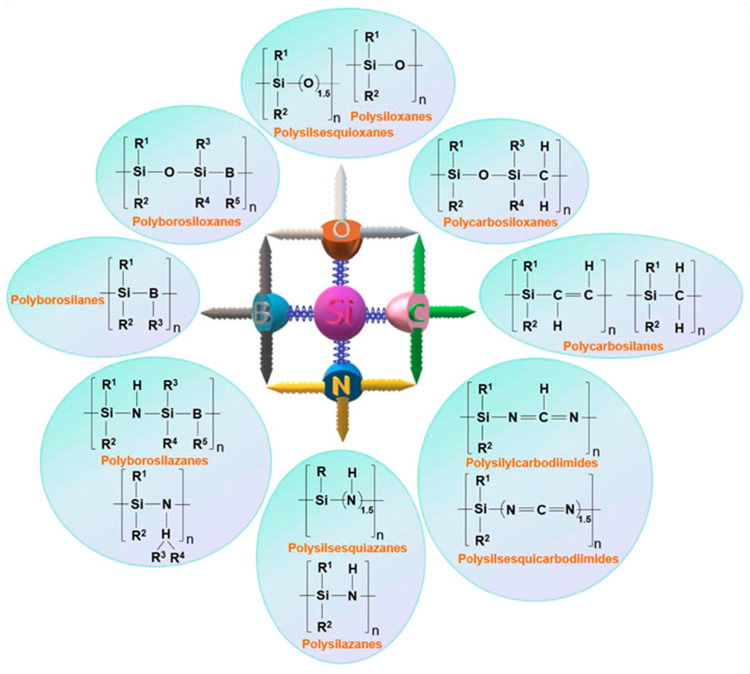
Different types of Si-based preceramic polymers (reprinted from [2] with permission from Elsevier).

The first step in the fabrication of Si-based polymer-derived ceramics is the synthesis of the proper organosilicon polymer. By grafting different elements such as oxygen, nitrogen, and carbon to the Si backbone structure, various types of Si-based polymers can be obtained [1,2], as shown in Figure 1. These polymers serve as precursors for producing a wide range of ceramic compositions, including SiC, SiO_2_, Si_3_N_4_, SiOC, SiCN, SiBCN, SiBOC, SiAlON, and other ceramics [2]. The most frequently used organosilicon polymers and their chemical formula, synthesis routes, and applications are listed in Table 1.

Only high-molecular-weight polymers that are capable of crosslinking can be used for the fabrication of polymer-derived ceramics [3]. A brief description of the important classes of preceramic polymers including polysilane, polycarbosilane, polysilazane, and polysiloxane are summarized below:

#### 1.1.1. Polysilanes

Polysilanes having a one-dimensional silicon backbone are basic precursors for the synthesis of SiC ceramics. Each silicon atom is attached to R_1_ and R_2_ substituents, whereas the substituents are presenting molecules with different combinations of C, H, S, N, and O atoms. Polysilanes are typically prepared using the method known as Wurtz-type coupling of halosilanes [12]. This synthetic approach involves the reaction of chlorosilanes with sodium or lithium dispersion, resulting in the reduction process that leads to the formation of polysilane. The reaction occurs within a high-boiling-point inert solvent, such as toluene, benzene, or tetrahydrofuran, under reflux conditions. Polysilanes have been proposed for various applications such as photoresists, photoconductors, and semiconductors [5]. Their σ-conjugation due to electron delocalization on silicon–silicon bonds results in distinctive optoelectronic and photoelectric properties. The properties of polysilanes stem from two primary factors: the side chain groups attached to the backbone and the molecular weight of the polymer [2].

#### 1.1.2. Polycarbosilanes

Polycarbosilanes with a Si-C backbone contain branched chains such as methylene, vinylidene, and phenylene which make them more complex compared to polysilanes [13]. The most common way to synthesize these polymers is thermal decomposition of polysilanes under pressure using the Kumada mechanism. However, the accumulation of decomposition gaseous products (e.g., methane, Me_3_SiH, SiH_4_) can lead to a dangerous increase in pressure in the autoclave. Synthesis of polycarbosilanes using pyrolysis of polydimethylsilane in a nitrogen atmosphere was performed at ambient pressure through catalytic processes (using polyborodiphenylsiloxane) in a reflux condenser [14]. This class of organosilicon polymers has been widely used for the fabrication of SiC-based components like fibers, composites, powders, and other non-oxide ceramic structures.

#### 1.1.3. Polysilazanes

Using polysilazanes with carbon containing side chains attached to a Si-N backbone, SiCN ceramics are synthesized. If a C-free polysilazane polymer like perhydridopolysilazane is utilized, Si_3_N_4_ ceramics can be produced. Fabrication of SiCN fibers from polycarbosilazanes was first demonstrated by Verbeek et al. in the 1970s [15] where oxidation resistance up to 1200 °C and a significant strength and elastic modulus were achieved.

Remarkable thermal stability, scratch resistance, and corrosion resistance and high hardness of polysilazanes make them ideal for various applications including protective and heat-resistant coatings in industries such as electronics, automotive, and aerospace as well as heat exchanger barriers [16] and for protecting steel against oxidation [17].

#### 1.1.4. Polysiloxanes and Polysilsesquioxanes

**Figure 2:**
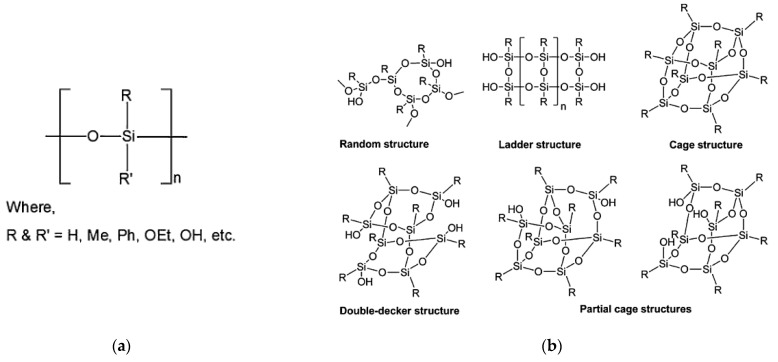
General structure of (**a**) polysiloxanes, and (**b**) polysilsesquioxanes (reproduced from [21] with permission from Springer Nature).

Polysiloxanes (Figure 2a) with a general formula of [R_2_SiO]_n_ are an inexpensive, widely used class of Si-based PCPs for the synthesis of silicone oxycarbide and oxynitride ceramics [2].

Equation (1) describes the formation of polysiloxanes through the reaction of chlorosilane precursors with water.

n Si(CH_3_)_2_Cl_2_ + nH_2_O → [Si(CH_3_)_2_O]_n_ + 2n HCl (1)

Ring opening polymerization of cyclic trimers and tetramers has been reported as a successful alternative to the hydrolysis approach [18]. Crosslinked polysiloxanes and polysilsesquioxanes can be obtained using the sol-gel process via hydrolysis and condensation reactions of organically modified silicon alkoxides, depending on their functional side chain.

Polysilsesquioxanes with a chemical formula of [RSiO_3/2_]_n_, as implied by their name, consist of a silicon atom (sil-), bonded to one and a half (-sesqui-) oxygen atoms (-ox-), and a hydrocarbon group (-ane) [19]. R can be either an H atom or an organic functional group such as methyl, phenyl, ethoxy, or hydroxyl. The nature of the organic ligands attached to the Si atoms determines the packing of the molecules and the intermolecular forces between them, which ultimately determine the physical state of the material. As a result, pure polysilsesquioxanes can be found in the form of liquid, crystalline, or amorphous powder. These hybrid organic–inorganic materials can have random, ladder, double-decker, cage, and partial-cage structures [20,21] (Figure 2b). Random structures are polymeric precursors that lack long-range order. The ladder ones consist of alternating silicon and oxygen atoms in an oligomeric scale arranged in a ladder-like structure. The process of hydrolysis and condensation of organosilanes results in the formation of partial-cage structures. These structures, despite being partially condensed, still have silanol groups present at one or more corners of the cage. Cage structures contain no silanol groups (-OH) as they are fully crosslinked.

Pyrolysis of polysiloxanes and polysilsesquioxanes results in the formation of siliconoxycarbide (SiOC) glasses [22]. Mechanical properties of carbon-rich SiOC glasses, particularly creep resistance, have been extremely improved compared to fused SiO_2_ [23]. The addition of ceramic or metallic particles allows for modification of the mechanical and physical properties of the SiOC matrix [24].

In addition to SiOC ceramics, silicate ceramics can be fabricated by introducing fillers to the polysiloxane/polysilsesquioxane polymers [1].

### 1.2. Properties of Si-Based Polymers

The unique properties of PCPs offer potential solutions for different applications where thermal stability is a necessity or the material experiences a harsh environment [5]. An intriguing aspect that encourages the utilization of PCPs is the possibility of modifying the functional groups within the starting precursor, thereby promoting its properties [2]. Using Si-based polymers, PDCs in the form of powders, fibers, coatings, composites, and complex three-dimensional (3D) structures can be produced. Diverse processing techniques have been implemented for fabrication of PDCs as they can be melted and crosslinked or dissolved in several solvents [2].

Due to their viscoelastic behavior above the melting point, and the miscibility with other polymers, various thermoplastic shaping methods can be utilized. Blending preceramic polymers with organic/inorganic additives facilitates the preparation of homogenous compounds for the fabrication of PDCs. Substituting PCPs for ceramic powders, it is feasible to mix the materials at lower temperatures, while also reducing abrasion within the machinery involved [25].

By dissolving PCPs in compatible organic solvents, mixed with other organic/inorganic additives, homogeneous ink, gel, or dried sediments (after drying) can be created. The obtained mixture can then be used for subsequent shaping or synthesis steps.

To fabricate polymer-derived 3D ceramic structures, organosilicon precursors need to have certain properties in order to be effective. One of these properties is the ability to crosslink. Following the shaping process, these polymers undergo a crosslinking stage to maintain the shape during subsequent thermal treatment [26]. Preceramic polymers with high molecular weight are advantageous as more crosslinks can form, resulting in a thermoset that has a high shape stability during the thermal debinding process [5]. Utilizing preceramic polymers to create PDCs, it is possible to do machining of the parts before ceramization [27]. In this way, the occurrence of brittle fractures during machining of the ceramic part can be prevented.

With pyrolysis and sintering of PCPs in either air or an inert atmosphere at relatively lower temperatures (800–1200 °C), a wide range of oxide and non-oxide ceramics can be obtained. Since the density values for the polymer (1–1.2 g/cm^3^) and ceramic phases (2–3.2 g/cm^3^) vary significantly, the resulting ceramic residue after pyrolysis may experience shrinkage of up to 70% in volume that leads to the development of considerable porosity or cracks. Therefore, achieving a ceramic yield above 60% is expected after pyrolysis [28]. Higher ceramic yield is more desirable for a preceramic polymer, resulting in less shrinkage and, subsequently, being less prone to defects, such as cracks and bubbles [29]. The release of gaseous products during crosslinking and pyrolysis of the PCPs is another factor that affects the ceramic yield and contributes to the formation of micro/macro porosity and cracks in the fabricated PDCs [26].

**Figure 3:**
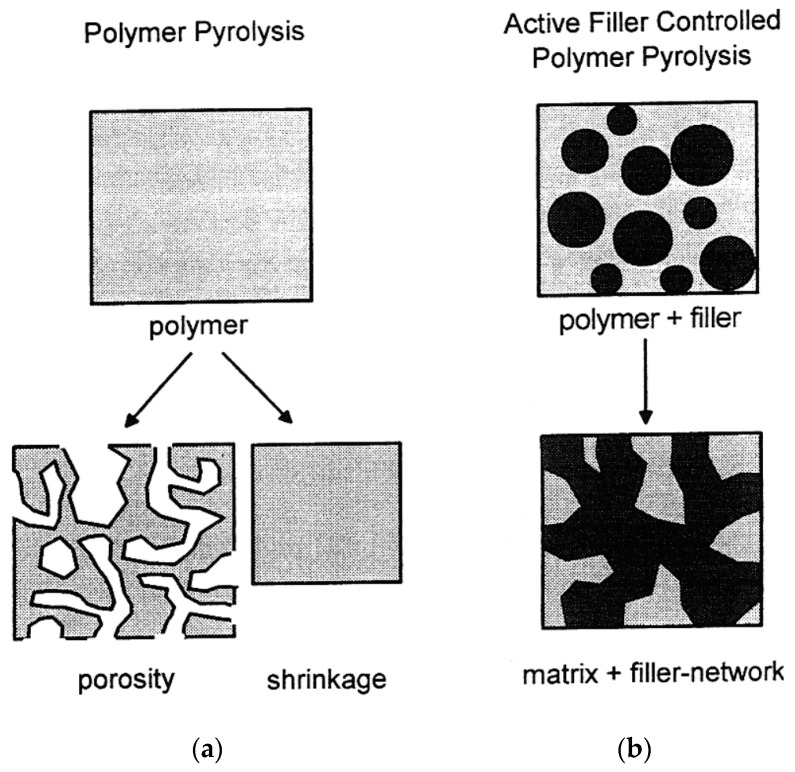
Microstructure evolution during polymer to ceramic conversion (pyrolysis step): (**a**) without and (**b**) with filler (reprinted from [32] with permission from Wiley).

To prevent the pore formation during crosslinking and pyrolysis, as well as the volume shrinkage of the PCPs during polymer to ceramic conversion [30], fillers have been commonly used for near-net-shape fabrication of PDCs by decreasing their freedom to shrink as was first introduced by Greil et al. in the 1990s [31,32] (Figure 3). By adding fillers to the PCP matrix, its mechanical, thermal, or other desired properties can be improved. The fillers are typically incorporated inside the matrix before the pyrolysis process. Depending on the specific application, desired characteristics, and compatibility with the PCP matrix, a wide range of fillers can be utilized [2,32].

**Figure 4:**
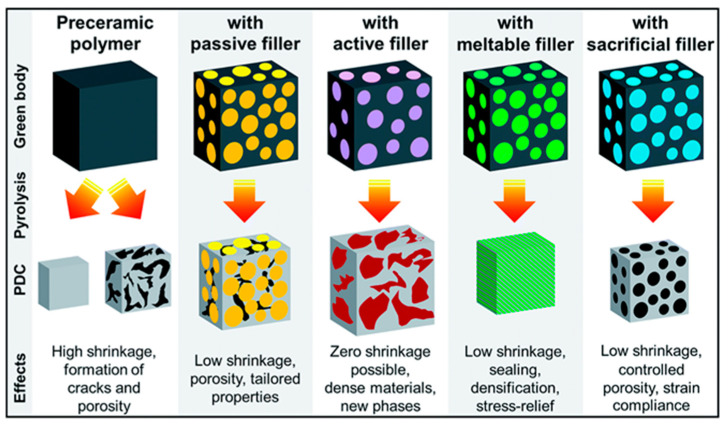
Different types of fillers and their application in fabrication of PDCs (reprinted from [33] with permission from the Royal Society of Chemistry).

Different shapes of fillers including powders, platelets, nanotubes, chopped and long fiber, and so on can be added to the PCP matrix before shaping. Fillers are divided into four groups as shown in Figure 4 [33]:

- passive fillers: this group of fillers is not reactive. They only control the shrinkage and presence of macro defects during pyrolysis. Typical examples are SiC and Si_3_N_4_.
- active fillers: using active fillers, on the other hand, a new phase compared to the starting PCP can be achieved. Carbides, nitrides, silicates, oxides, and silicides can be produced as a result of a reaction between the filler and atmosphere or PCP residue after pyrolysis or the gaseous products during pyrolysis itself.
- meltable fillers: this category of fillers consists of meltable materials, typically glasses. When subjected to high temperatures, the glass phase melts or softens and effectively fills the available porosity which enhances the density. This approach protects the part against oxidation and corrosion. When meltable fillers are used in coatings, their softening at elevated temperatures reduces Young’s modulus, allowing for the relaxation of thermomechanical stresses arising from mismatches in thermal expansion between the substrate, coating, and fillers within the precursor matrix. They may also undergo chemical reactions with other components in the system, acting as active fillers.
- sacrificial fillers: These organic compounds are mixed with the PCP and removed after crosslinking using thermal decomposition or dissolution in a solvent. Their main function is to create the porosity in the PDC parts.

The choice of fillers depends on the desired properties of the final ceramic material and the compatibility with the preceramic polymer matrix. The amount and dispersion of fillers within the polymer matrix also play a crucial role in determining the overall performance of the resulting ceramic material.

Ceramic powders such as silicon carbide (SiC) [34], silicon nitride (Si_3_N_4_) [35], alumina (Al_2_O_3_) [36,37,38], and boron nitride (BN) [39] can be added to create composite or new phases after pyrolysis and enhance the mechanical properties. Using carbon-based fillers like carbon fibers and carbon nanotubes (CNTs) has been employed to improve the mechanical properties, electrical conductivity, and thermal stability of the preceramic polymers [26,40]. Incorporating metallic powders or particles, mixed with a PCP as a reactive binder, PCP can improve wear resistance and corrosion resistance properties of the metal part [26]. The addition of fillers such as molybdenum disilicide (MoSi_2_) [41] and iron silicide (FeSi) can be introduced to adjust electrical conductivity or magnetic properties. Polymeric fillers like cellulose fibers can be used as fillers to modify the rheological properties, processability, or combustibility of the preceramic polymer [42]. Also, Kim et al. in 2005 used poly (methyl methacrylate-co-ethylene glycol dimethacrylate) microbeads to create partially interconnected microcellular ceramics [43].

Due to the importance of the manufacturing process of oxide and silicate 3D ceramic structures using polysiloxane/polysilsesquioxanes through the material extrusion-based additive manufacturing (MEX-AM) technique, the following sections mainly focus on the synthesis of oxide and silicate ceramics.

### 1.3. Synthesis of Silicate Ceramics Using Polysiloxane and Polysilsesquioxanes

In recent years, there has been a lot of interest in polymer-derived silicate ceramics by incorporating active fillers into the PCP matrix. As can be seen in Table 2, various types of silicate ceramics have been successfully produced using polysilsesquioxanes. It should be noted that the fabrication of silicates using organosilicon polymers requires handling and heat treatment in an air atmosphere [38,44,45]. Pyrolysis of PCPs in different atmospheres alters the ultimate composition. Using air atmosphere results in a SiO_2_ residue, whereas working under an inert atmosphere (e.g., nitrogen or argon), silicon oxycarbide (SiOC) and residual carbon composition can be achieved [1,46,47].

In 2006, Bernardo et al. [37] reported the use of nano-fillers to enhance the reactivity between the PCP and the active filler. Their study employed a mixture of methyl-silsesquioxanes, namely SILRES MK (MK), and γ-Al_2_O_3_ (average particle size of 15 nm) to produce dense 3:2 mullite disks (d ≈ 31 mm, h ≈ 5 mm). At a temperature of 1250 °C, mullitization, with a volume fraction of 96%, was already observed. This percentage increased to 99.5% at temperatures exceeding 1350 °C.

By sintering at 1550 °C for 2.5 h, complete mullitization was achieved, with only a remaining volume fraction of 0.1% silicate glass. The presence of residual cristobalite was previously reported [36,66,67]. The absence of cristobalite impurity in this study was attributed to the effective dispersion of highly active nano-alumina within the PCP matrix. Colombo et al. reported the fabrication of different silicate ceramics using nano-active fillers [25]. Pure mullite, zircon, cordierite, forsterite, and yttrium-silicates with low grain size were achieved at low sintering temperatures and dwell times as a result of highly favorable reaction kinetics. However, the issue with densification in the polymer-derived silicates persists due to the typical poor ionic interdiffusion in silicates. Therefore, submicron pores appear between densified areas.

**Figure 5:**
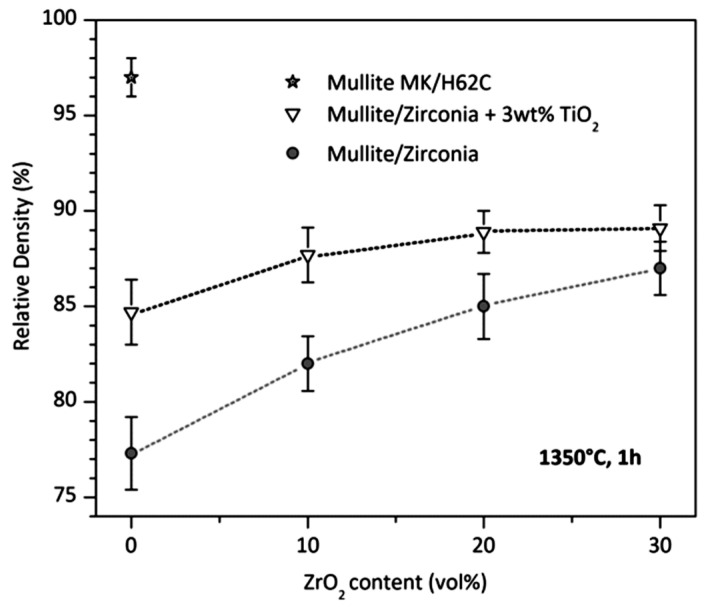
Fabrication of mullite-based ceramics from MK polymer filled with nano-γ-Al_2_O_3_: the impact of incorporating secondary filler (ZrO_2_), sintering additive (TiO_2_), and partial modifications in the starting polymer composition (mixing MK and H62C) [1]. Open access CC-BY.

Figure 5 illustrates the porosity values for pure mullite derived from MK and nano-sized γ-Al_2_O_3_ (more than 20%). By incorporating a secondary inert filler (i.e., ZrO_2_), zirconia-toughened mullite with higher density was obtained. The addition of secondary fillers reduces the amount of PCP. In this way, fewer gaseous products are formed during PCP transformation to PDC and fewer pores are generated. Although the porosity is reduced to 15% or less, the material remains porous. Introducing TiO_2_ as a sintering additive, transient viscous sintering occurs (above 1200 °C) that enhances the sinterability of the mullite phase as well as zircon (ZrSiO_4_) [53] and forsterite (Mg_2_SiO_4_) [54].

Substituting a fraction of MK for SIRLES H62C, a relative density of 97% was achieved. The authors explained this observation by varying levels of network connectivity and defects in the amorphous silica phase of different organosilicon polymers, as a more defective silica network is favorable for accommodating the Si-O network containing fragments into the mullite structure [1].

The same approach has been successfully used to prevent the extensive cracking of zircon [53], forsterite [54], and cordierite [55] ceramics. Partial replacement of MK with SILRES H62C (H62C) enhanced the compactness due to the different molecular arrangement of the PCP, leading to diverse possibilities to rearrange the molecular structure. Also, H62C goes through different crosslinking reactions that avoid gas release during pyrolysis. In this way, local pressure accumulation from generated gaseous products is eliminated. A crosslinking step (at 250 °C for 30 min) prior to shaping can be beneficial due to a certain amount of gas release and shrinkage before the pyrolysis step.

**Figure 6:**
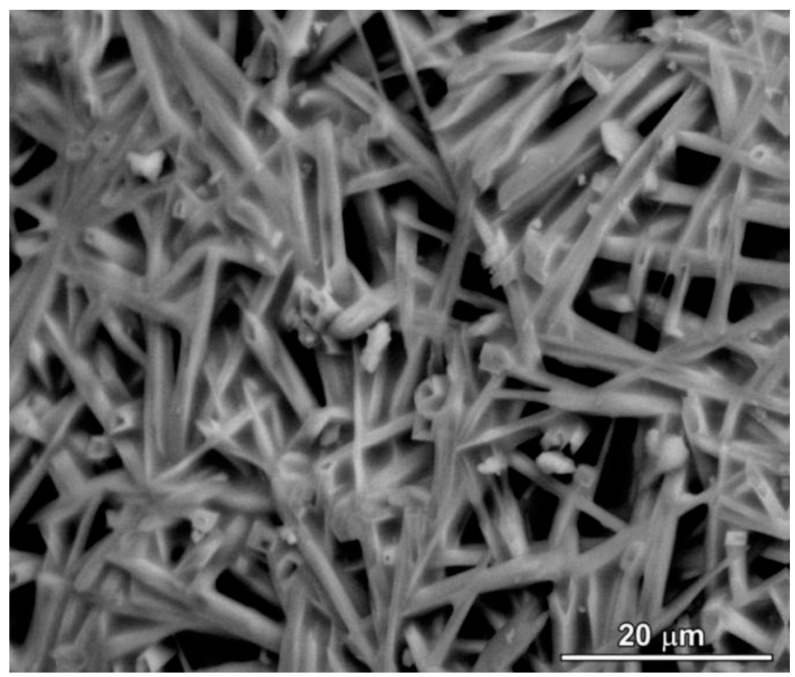
Microstructure of polymer-derived mullite ceramic, based on the mixture of MK, nano-γ-Al_2_O_3_, and borax [1]. Open access CC-BY.

The microstructure of alumina-rich polymer-derived mullite ceramics (>74 wt% Al_2_O_3_) typically exhibits equiaxed grain morphology due to limited kinetics in solid-state cation interdiffusion. It has been demonstrated that the addition of B_2_O_3_ can shift the mullitization to lower temperatures and encourage the anisotropic grain growth by lowering the viscosity of the intergranular phase [68]. Bernardo et al. demonstrated the fabrication of acicular mullite fibers by adding 3 wt% hydrated sodium borate to the mixture of MK and nano-γ-Al_2_O_3_ [1] (see Figure 6). Such interlocking grown fibers can be a candidate for the fabrication of highly porous materials. Acicular mullite ceramics, which are typically produced through complex thermo-chemical processes, can be replaced with the polymer-derived cellular ceramics [69].

To improve the relative density of mullite-based ceramics from 93% to 97%, Riedel et al. used an MK polymer filled with functionalized nano-γ-Al_2_O_3_ (added octylsilane groups on the surface) [70] and obtained a high amount of mullite crystals already at 1300 °C, attributed to the better distribution of functionalized γ-Al_2_O_3_ within the initial PCP compared to unfunctionalized fillers. Due to heat treatment of the mixture in the nitrogen atmosphere, silicon oxycarbide (SiOC) was yielded from MK, which transformed into SiC crystals due to the interaction with γ-Al_2_O_3_. The resulting SiC-mullite monoliths can be an alternative for SiC/mullite nanocomposites derived from polymethylsiloxane gels filled with α-Al_2_O_3_. The obtained crack-free monoliths can be potential candidates for complex-shaped ceramics, resistant to high temperatures and corrosion. A ternary SiC-mullite composite containing nano-α-Al_2_O_3_ crystals was obtained by substituting nano-aluminum fillers for γ-Al_2_O_3_ [71].

In binary systems consisting of silica and a metal oxide, the formation of multiple silicate phases requires having a specific molar ratio between silica and the metal oxide. For instance, when silica is combined with CaO, various silicates such as CaO·SiO_2_, 3CaO·2SiO_2_, 2CaO·SiO_2_, and 3CaO·SiO_2_ can be produced depending on the CaO/SiO_2_ molar ratios. These silicates can be mainly used for biomaterial applications [49]. Using a filler like CaCO_3_ can result in the formation of a silicate with a higher CaO/SiO_2_ ratio than intended. For example, when micro-sized CaCO_3_ is used in a filler/PCP formulation, the desired wollastonite (CaO·SiO_2_) phase is replaced with di-calcium silicate (2CaO·SiO_2_) due to localized concentrations of CaO. On the contrary, almost pure wollastonite was obtained using nano-sized CaCO_3_ under the same conditions [48].

The choice of PCP has been found to influence the polymorphism of calcium silicates. Using H62C with a low molecular weight instead of MK, a trace of the α-phase or “pseudowollastonite” was detected alongside the β-phase typically obtained at the temperature range of 900–1100 °C [49]. The polymer with a lower molecular weight consists of short Si-O fragments that promote the formation of ring-structured silicate variants like the α-phase [72].

Synthesis of binary ceramic compositions such as forsterite and yttrium silicates has been reported as well. A highly reactive combination of PCP filled with nano-sized MgO enables the formation of forsterite for biomaterials [73] and dielectric applications [74] at temperatures as low as 800 °C. Biocompatibility and bioactivity observed in forsterite monoliths [75] and coatings [76,77] have made them a promising material for medical application. Moreover, forsterite has a low dielectric constant (ε_r_ = 6.8) which makes it attractive for submillimeter-wave applications. The low dielectric losses and the ability to transfer signals in a short time are interesting for high-frequency electromagnetic waves. This makes it a desirable choice for dielectric resonators, filters, ultra-high-speed LAN, and car anti-collision systems [78]. Despite its interesting characteristics, forsterite possesses limited ionic interdiffusions, which result in poor solid-state sinterability [79]. As a result, the presence of unreacted MgO or formation of Mg-silicates with a varying MgO to SiO_2_ ratio is possible; therefore, enstatite forms in MgO-poor regions [80]. The inclusion of nano-sized TiO_2_ not only improves densification (by forming Mg_2_Si_0.9_Ti_0.1_O_4_ solid solution), but also plays a crucial role in eliminating unreacted MgO and enstatite impurities [54].

A combination of yttria (Y_2_O_3_) and silica (SiO_2_) can produce mono- and di-silicates such as Y_2_O_3_·SiO_2_ (or Y-MS) and Y_2_O_3_·2SiO_2_ (Y-DS) [52]. Y-MS is monophasic, undergoing a dislocative transformation between the X1 (low temperature) and X2 (high temperature) phases. Y-DS, however, exhibits multiple polymorphs (y, α, β, γ, δ, z), with each form stable within a specific temperature range. Depending on the processing, different polymorphs can be obtained [81]. The sol-gel method encourages the formation of the α-phase [82,83], while hydrothermal synthesis enables the development of the y-phase [84]. Mixing MK with nano-Y_2_O_3_, it is possible to achieve both silicates at lower temperatures (1000–1350 °C) [52].

Synthesis in ternary systems is quite challenging due to the potential formation of multiple silicate phases resulting from recombination of silica and various oxides in different ratios. For instance, in the CaO-MgO-SiO_2_ system, both diopside (CaMgSi_2_O_6_) and akermanite (Ca_2_MgSi_2_O_7_) can form. In the case of cordierite (2MgO·2Al_2_O_3_∙5SiO_2_) and gehlenite (2CaO·Al_2_O_3_·SiO_2_), the presence of silica-free compounds such as Mg and Ca aluminates, respectively, is probable. The presence of such silica-free phases hinders the full interaction between oxides and polymer-derived silica, increasing the risk of residual SiO_2_ in the final product. To address this issue, the firing temperature is modified. For example, firing polymer-derived cordierite at 1350 °C can eliminate the MgAl_2_O_4_ phase. Alternatively, the ionic interdiffusion was promoted by generating solid solutions with more “open” crystal structures. An example of this approach involves the partial substitution of Ca^2+^ ions with Eu^3+^ ions to produce luminescent materials. Formation of Ca_2−2x_Eu_2x_Al(Al_1+2x_Si_1−2x_O_7_) solid solutions by adjusting the Al/Si ratio can significantly reduce the Ca-aluminate content [85].

### 1.4. Processing of Preceramic Polymers

**Figure 7:**
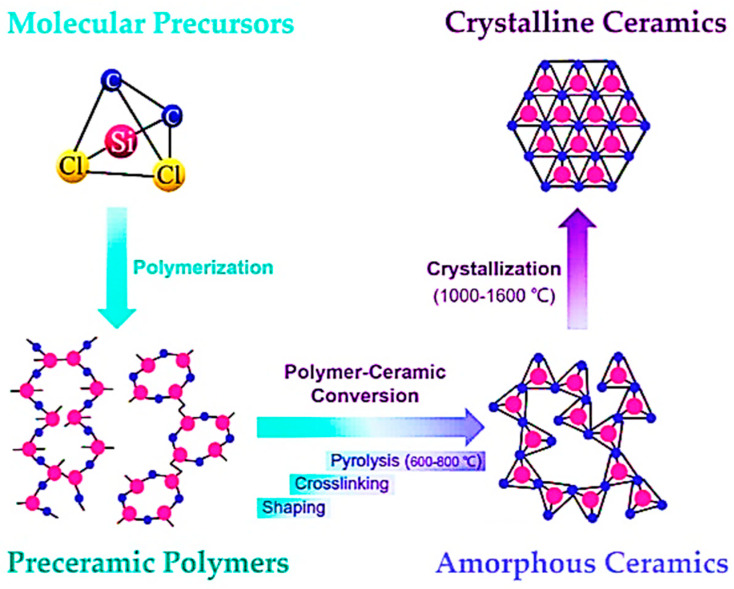
Processing steps for fabrication of PDC components, from shaping and crosslinking to pyrolysis and crystallization (sintering) (reprinted from [2] with permission from Elsevier).

Using preceramic polymers to fabricate 3D ceramic structures, several steps need to be taken including shaping the material, followed by crosslinking and pyrolysis. After that, a sintering step can be implemented if necessary. Figure 7 demonstrates the necessary processing steps to achieve polymer-derived ceramic (PDC) components.

#### 1.4.1. Shaping

One of the advantages of PCPs is their viscoelastic behavior rising from their polymeric nature. As a result, PCPs are well-suited for a wide range of thermoplastic shaping techniques such as casting, injection molding, pressing, tape casting, extrusion, fiber drawing, and coating [26]. More recently, using additive manufacturing (AM) techniques for shaping PCPs attracted interest among researchers as well. This topic will be discussed in more detail in Section 2.2. Utilizing PCPs also eliminates the concerns from powder-based and sol-gel approaches. They prevent drying issues and long processing times for gelation and drying as well as maintaining constant rheological properties within the processing time window. PCPs also minimize the need for flammable solvents and special handling processes. Furthermore, their solutions remain stable for extended periods and fillers can be easily incorporated to produce composite ceramics.

#### 1.4.2. Crosslinking

Crosslinking is the process by which polymer chains are linked together, forming a three-dimensional network. This process is important for almost all the fabrication processes of PDC structures because it allows the precursor to transform from a flexible, thermoplastic organic material (necessary for shaping) into a rigid and insoluble thermoset and preserve the structure during post processing [20]. Crosslinking can occur through a variety of mechanisms, most commonly by thermal crosslinking in the range of 100–250 °C under airflow, crosslinking with chlorosilane, or curing with radiation [30]. With the presence of functional groups such as Si-H, Si-OH, and Si-vinyl, PCPs can be spontaneously crosslinked below 200 °C using hydrosilylation (addition) or silanol–silanol reactions (condensation) [26]. Crosslinking in an air atmosphere has traditionally been a popular method for curing PCPs to provide SiO_2_ yield. This process, however, is not suitable for the fabrication of non-oxide ceramics due to the drawback of leaving up to 15 wt% oxygen in the final PDC [86,87,88]. This residual oxygen content can adversely affect the thermal stability of the non-oxide PDCs, weakening their overall performance.

The utilization of a catalyst offers the advantage to crosslink the preceramic polymers at lower temperatures. The early linking of the volatile oligomers, facilitated by the catalyst, contributes to shape stability. Because of the crosslinking, the volatile oligomers which typically evaporate or degrade are effectively incorporated into the crosslinked network and increase the yield of the obtained ceramic [88].

Crosslinking using UV radiation is applicable when PCP contains photosensitive functional groups in its backbone structure. This method can be mostly effective when utilized for crosslinking preceramic polymers in the form of fibers or thin structures due to its limited penetration depth [89,90]. Another common crosslinking mechanism called “free radical polymerization” is utilized for the vat photopolymerization additive manufacturing (AM) technique. This method involves the absorption of UV photons with a photoinitiator (PI). This process generates highly reactive free radicals (R*) that react with the monomer (M), leading to the formation of polymer chains and the progression of the polymerization process.

For pure commercial methyl-hydroxyl-siloxane and methylphenyl-hydroxyl-siloxane PCPs, Danko et al. investigated microwave heating to obtain SiOC ceramics [91]. They reported that using fast microwave heating was not feasible as intensive mass loss from rapid crosslinking and pyrolysis of samples resulted in bloating or cracks. Because of low conductivity and poor dielectric properties, microwave heating is challenging, especially during the early curing stages [92]. Therefore, microwave heating does not seem to be a promising processing method for curing pure PCPs. Evaluation on a case-by-case basis should be performed and further investigation is required to discover the potential of this method.

#### 1.4.3. Pyrolysis

Shaping and crosslinking of PCPs is followed by a pyrolysis step, known as ceramization, in which the PCP is transformed into a ceramic material through various thermal processes. These processes include hot pressing, spark plasma sintering, chemical vapor deposition, plasma spraying, rapid thermal annealing, laser pyrolysis, microwave heating, and the most commonly used method, pyrolysis in an argon or nitrogen atmosphere. Pyrolysis in such inert atmospheres leads to the formation of amorphous covalent ceramics with the decomposition of organic side chains (methyl/phenyl/vinyl groups) or Si–H, Si–OH, or Si–NH_x_ groups and the gradual removal of the gaseous byproducts at elevated temperatures (600–1000 °C). The reaction mechanisms during pyrolysis have been studied using solid-state nuclear magnetic resonance (NMR), Fourier transform infrared spectroscopy (FTIR), Raman spectroscopy, and a thermogravimetric analysis (TGA) coupled with mass spectroscopy. The use of ion irradiation to eliminate hydrogen atoms through the cleavage of C-H bonds is an alternative non-thermal process for ceramization where any remaining carbon is transformed to diamond-like carbon clusters. The pyrolysis atmosphere can modify the composition of the final ceramic component. Pyrolysis of a preceramic polymer under an inert atmosphere (argon or nitrogen) is required to create SiC, Si_3_N_4_, SiCN, SiBC, or SiCBN (depending on the starting PCP composition) whereas pyrolysis in air can be employed for the formation of silicate ceramics using active metal oxide fillers.

During the ceramization process of PCPs, important parameters such as heating rate, reaction atmosphere, reaction temperature, and dwell time influence the phase composition and microstructure of the final ceramics. They influence the extent of crystallization, carbothermal reduction reactions, and filler reactions within the material.

Pyrolysis of polysiloxane/polysilsesquioxanes in an air atmosphere has been widely used for the fabrication of oxide ceramics using active fillers. The following section reviews the studies employing pyrolysis in an air environment.

## 2. Additive Manufacturing of Preceramic Polymers

The versatility of polymer-forming technologies allows for shaping preceramic polymers in various ways and thereby overcoming the limitations and drawbacks associated with a traditional ceramic shaping process like pressing, extrusion, or injection molding. The pressing method, followed by pyrolysis and sintering, is an effective method to produce PDCs without or with very low content of porosity. However, it is limited to simple shapes. When this method is employed for more complex structures, a subtractive machining step is necessary, which is expensive and time-consuming, and results in significant material waste [93]. To fabricate more complex structures, extrusion and injection molding have been employed [94]. Nevertheless, the extrusion process is limited to circular symmetric objectives and for the injection molding, designing of molds is required, which makes prototyping economically unreasonable due to the cost of the molds. Even though the complexity of ceramic parts increases from pressing to injection molding, additive manufacturing (AM) has the highest freedom of geometrical design. Using AM, a 3D computer model with complex and tiny geometry can be converted into a three-dimensional (3D) structure by depositing the material layer by layer. In this way, even dense structures with controlled inner porous structure designs can be achieved easily without using pore formers. In addition, the need for costly molds and material waste can be avoided. A summary of different AM methods used for the fabrication of PDCs with polysiloxane/polysilsesquioxane is presented in Table 3.

### 2.1. Light-Assisted AM (Vat Photopolymerization)

Vat photopolymerization techniques including the first invented stereolithography (SL) method [96] followed by digital light processing (DLP) and two-photon polymerization (TPP) as derivatives of the SL technique can provide accurate 3D replicas with fine details and utilize similar principles but have some key differences in their implementation [97].

PCPs are highly compatible with AM involving vat photopolymerization [98]. In SL of PCPs, precise patterning of PCPs and subsequent conversion to ceramics through pyrolysis result in complex PDCs with a high resolution. The PCP needs to be photosensitive, soluble in compatible solvents, and providing proper rheological behavior. Ensuring a homogeneous dispersion of PCPs in the liquid phase also helps minimize scattering, resulting in a high-quality surface finish for the final PDC components.

A general description of these printing methods and the existing research on using SL and DLP for manufacturing parts with polysiloxane/polysilsesquioxane materials are provided in the following:

#### 2.1.1. Stereolithography (SL)

Stereolithography (SL) enables the creation of precise objects with defined edge quality. The SL method is based on photopolymerization of liquid resins containing monomers and oligomers that are sensitive to a specific wavelength of light [99]. These so-called photopolymers undergo a chemical reaction using exposure to a specific wavelength and the liquid transforms into a solid polymer. In this process, a thin layer of the photopolymer is deposited on the build platform. This layer of resin is exposed to a specific pattern of light, typically ultraviolet (UV) light, using a point-by-point scanning method, where the laser beam sequentially cures the resin. The photoinitiator in the resin absorbs the light energy and cures the resin in the defined areas to form a solid layer. After curing one layer, the platform moves down to create space for the next layer of liquid resin. Each cured layer bonds to the previous layer, creating a cohesive structure. This process continues layer by layer until the desired 3D structure is built. After printing, the printed form is treated with the proper solvent to remove the extra uncured resin. Using the SL technique for 3D printing of PCPs, a high printing resolution (20 μm or less) can be achieved [100]. However, the full potential of this method is still not fully explored due to a limited number of proper photocurable PCPs.

Ożóg et al. in 2022 fabricated highly porous gyroid scaffolds obtained with masked stereolithography [101]. In this study, Biosilicate^®^ glass–ceramics were produced using a phenyl silicone resin (H44)/photocurable liquid acrylate blends at a ratio of H44/SB/IPA = 1/1/0.5. Calcium carbonate (CaCO_3_), sodium carbonate (Na_2_CO_3_), and sodium phosphate (Na_4_P_2_O_6_) were used as the fillers. Pyrolysis was performed in a nitrogen atmosphere (0.5 °C/min up to 500 °C for 5 h, followed by heating at 2 °C/min up to 1000 °C for 1 h).

Rosado et al. in 2023 reported utilizing a commercial silica polyacrylic resin and nano-Al_2_O_3_ to produce mullite-based scaffolds using stereolithography [102]. One approach involved infiltrating a colloidal alumina sol into the printed porous silica components. A second more effective method follows the printing silica/alumina components using a photocurable alumina resin mixed with the silica resin. The presence of higher alumina concentration and larger reaction surface result in a higher degree of mullitization in the final parts.

SiOC microlattice and honeycomb cellular structures were fabricated in a notable work by Eckel et al. in 2016 [103]. A combination of (mercaptopropyl) methylsiloxane and vinylmethoxysiloxane was printed after the addition of a UV free-radical photoinitiator, free-radical inhibitor, and UV absorber. Subsequently, samples went through a pyrolysis step at 1000 °C in an argon atmosphere. Final SiOC amorphous structures were dense and defect-free with only 30% linear shrinkage. The obtained cellular SiOC microlattices in this study demonstrate a 10 times higher compression strength in comparison to commercial SiC (Duocel) or silicon oxycarbide and aluminosilicate foams with similar density [103]. Furthermore, they display the ability to withstand temperatures as high as 1700 °C in oxidative atmospheres. SiOC foams are reported as potential candidates for high-temperature applications (e.g., core of load-bearing ceramic sandwich panels) or hypersonic vehicles and jet engines.

Although complex structures with fine details are produced with the SL method, due to the discrete layer-by-layer photopolymerization process, obstacles such as anisotropy in the shape and mechanical properties have been observed. The nature of the SL method reduces the printing speed and can cause a staircase effect in the printing sample. By introducing slant beam rotation (SBR) scanning, Aerif et al. in 2009 suggested a different approach to reduce the roughness in the stair stepped object [104]. In this method, a UV light beam is angled to create slanted edges within each layer and add an extra degree of freedom in the scanning mechanism to rotate 360 degrees inside the resin.

#### 2.1.2. Digital Light Processing (DLP)

The digital light processing (DLP) technique shares similarities with SL. However, it uses a different approach to achieve layer-by-layer printing [105]. In DLP, the light source is a digital projector with a digital micromirror device (DMD) that projects a patterned image of an entire layer onto a vat (the container of liquid resin) that allows for higher resolutions without the need for complex scanning processing. That is why DLP offers a higher printing speed compared to SL since the whole layer can be exposed to light simultaneously. Here, a photopolymer resin sensitive to light, specifically to a certain wavelength such as ultraviolet (UV) or visible light, is used. To start the printing process, the build platform is lowered into the vat, submerging a thin layer of liquid resin. The digital projector then projects the patterned image onto the resin surface. Selective curing of the resin happens in the illuminated areas. The exposed resin undergoes photopolymerization, transforming from a liquid to a solid state to create the desired pattern. The platform is then incrementally raised or lowered to deposit a new layer. The process is repeated until the 3D form is completed. Then, the printed object is typically removed from the vat and rinsed to remove any excess or uncured resin.

**Figure 8:**
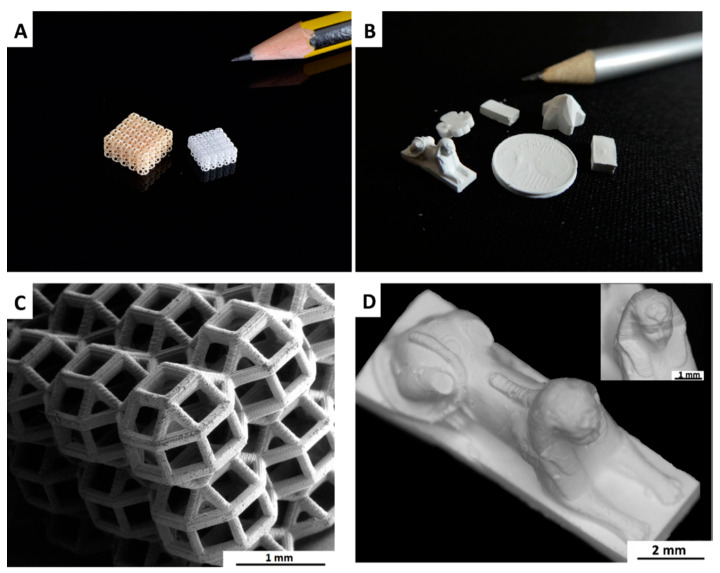
Complex porous structure fabricated with DLP before (**A** left) and after (**A** right, **C**) sintering, and bulk parts (after sintering (**B**,**D**)). The coin in Figure 8B was manufactured with soft lithography. (reprinted from [45] with permission from Elsevier).

Fabrication of complex mullite structures (Figure 8) using the DLP method was reported by Schmidt et al. in 2019 [45]. A mixture of highly acrylated, liquid photocurable polysiloxane (TEGO RC711) and nano-γ-Al_2_O_3_ was printed and cured with a UV lamp for 2 min. Samples were debound by heating up to 500 °C at 1 °C/min to decompose the polymer network and then fired between 1300 and 1400 °C to produce a 3D-printed mullite structure. Using nano-γ-Al_2_O_3_, pure mullite was achieved at 1300 °C. By increasing the filler size to micron-sized γ-Al_2_O_3_, the remaining alumina phase was detected.

Although the authors showed more desirable mullitization using nano-fillers, it was found that the suspensions containing nanoparticles had some drawbacks for 3D printing [45]. A higher amount of solvent was used for nano-fillers, which resulted in a low ceramic yield and the formation of a weak polymerized acrylate network. Due to these reasons, printed samples suffered from low printing resolution, cracking, and structure collapse during sintering. This issue was solved by employing micro-sized alumina powder to achieve dense and crack-free bulk components, as well as porous components with complex shapes. The porous components exhibited a total porosity of 90 vol% and compression strength of 1.8 ± 0.3 MPa.

Fabrication of different bioactive scaffolds has been reported [106]. Dasan et al. fabricated akermanite scaffolds [106] using H44 or H62C silicone resin. In addition, they investigated the effect of different fillers, such as CaCO_3_ powder, Mg(OH)_2_ micro-powders, nano-MgO powder, and borax. After printing, samples were sintered up to 1100 °C using a stepwise heat treatment. Using 4.5 wt% Na_2_B_4_O_7_ borax inside the compound with an H44 polymer, crack-free and phase-pure scaffolds with microporous struts were created. The compressive strength of a scaffold with this composition was comparable with the values for silicate scaffolds having the same porosity level [107].

**Figure 9:**
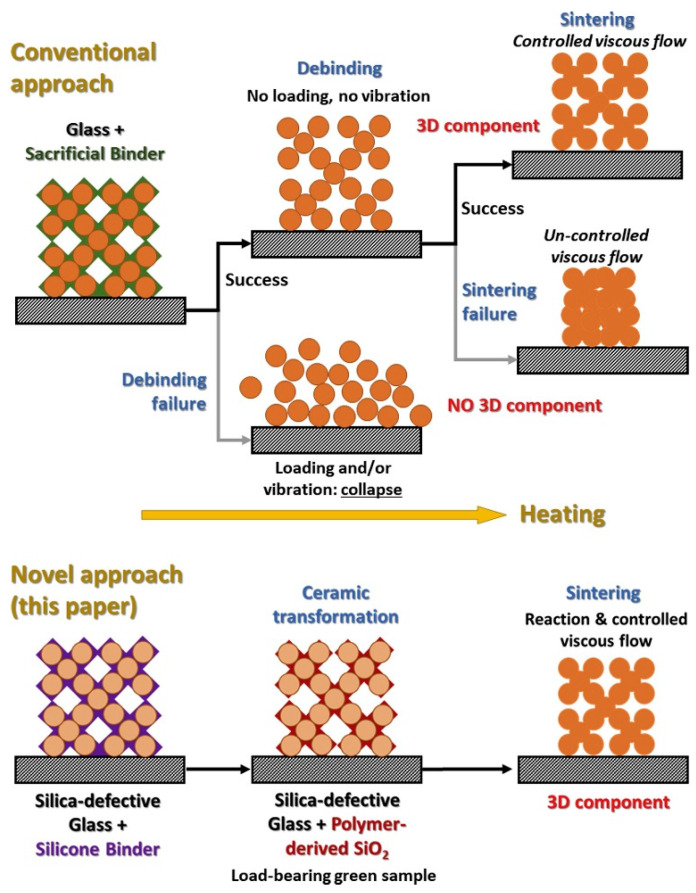
Fabrication of glass ceramic scaffold with conventional method and by using silica-defective glasses. (reprinted from [106] with permission from Elsevier).

Crack-free glass–ceramic scaffolds have been developed for use in tissue engineering applications [106] as shown in Figure 9. A mix of glass powders containing WB (indicating the crystalline phase; W and B are referring to wollastonite and calcium borate, respectively) and a ‘silica-defective’ variant, WB-15, was mixed with MK (responsible for binding the glass powder) and was printed. Ceramization of MK at elevated temperatures generates an amorphous silica phase up to 800 °C. Formed silica promotes shape stability even at sintering temperatures when glass powders are softened.

Ultrathin (with a thickness of 1.5 mm) scaffolds, containing 60 vol% porosity, were fabricated by Wang et al. for orbital bone repair via the DLP method [108]. The dilute magnesium-substituting wollastonite (CSi-Mg) scaffolds showed strong mechanical strength and stable biodegradability under a relatively low sintering temperature. The obtained scaffold with CSi-Mg composition was proposed as a good candidate for osteoconduction.

He et al. printed zirconia/calcium silicate (CS) composite scaffolds using the DLP method [109]. After sintering, the calcium silicate phase was embedded between the ZrO_2_ grains. The presence of the CS phase improved the cell proliferation and differentiation of the ZrO_2_ ceramic. Degradation of the scaffold resulted in the deposition of apatite, which was favorable for the integration of the scaffold with living bone. The obtained ZrO_2_/CS composite scaffold can be a good candidate for 3D printing bone repair scaffolds due to their enhanced biocompatibility compared to the ZrO_2_ scaffold.

#### 2.1.3. Two-Photon Polymerization (TPP)

Using two-photon polymerization (TPP), high-resolution, complex three-dimensional micro to nanoscale structures can be obtained [110]. In this method, a photosensitive material absorbs a femtosecond laser beam in the near-infrared range with a high photon energy density. The material molecules enter the excited state at irradiated locations and create a voxel (volume pixel) with the two-photon absorption process [111]. In this way, each layer is built by curing the photopolymer point by point until the full 3D form is obtained. Using this method, the fabrication of unique 3D structures with two-photon polymerization is an ideal technique for various applications in fields such as microelectronics, photonics, biomedicine, and other areas that require precise fabrication at the micro- and nanoscale. These applications encompass tissue-engineered scaffolds, micro medical devices like micro swimmers and needle arrays, optical devices, and more [111]. Using the TPP method, only SiOC ceramics were obtained [103,112,113].

A hybrid approach employing vat photopolymerization additive manufacturing (AM) methods has emerged to enable the fabrication of hierarchical PDC structures with varying resolutions. The first attempt to use a hybrid AM approach for PDCs was reported by Schmidt et al. in 2019 [114] to fabricate SiOC woodpile structures. A combination of digital light processing (DLP) and two-photon lithography (2PL) techniques has been employed to create complex SiOC ceramic components at different length scales. Submicron surface features were printed on top of the DLP-printed woodpiles with the 2PL method. In this way, highly precise microstructures with dimensions exceeding the limitations of DLP technology were successfully made. Nonetheless, the use of two PCPs, namely modified MK and photosensitive TEGO^®^ RC 711, revealed a challenge because of the shrinkage mismatch after pyrolysis. A solution was found by exclusively using RC 711 siloxane acrylate resin, ensuring uniform and consistent shrinkage. These fine detailed structures hold promise for the fabrication of micro-needles, micro-nozzles, and MEMS.

### 2.2. Selective Laser Sintering (SLS)

Using the selective laser sintering (SLS) technique, a laser beam facilitates the fusion or sintering of powdered materials together to build 3D structures [115]. First, a thin layer of powder is deposited on the build platform. A high-powered laser beam is exposed on the powder bed, accurately tracing the desired object shape. Consequently, particles stick together because of the material’s partial melting and selective fusion in defined areas. After sintering each layer, the platform is lowered to prepare a new layer of powder for sintering. Once the sintered 3D structure has undergone cooling, it is taken out for subsequent processing, e.g., remove the excess powder and finish the surface followed by heat treatment, depending on the material and desired specifications.

The research from Friedel et al. in 2005 is the only reported study on using SLS specifically for curing PCPs [116]. They referred to this process as selective laser curing (SLC). Poly(methylsilsesquioxane) (MK) mixed with a SiC passive filler was SLS printed and cured using a CO_2_ laser beam at 400 °C. MK was cured due to a condensation reaction between hydroxyl and ethoxyl groups, transforming the resin from a thermoplastic resin into a thermosetting material. An additional advantage of working with molten MK is its ability to blend uniformly with fillers, facilitating the formation of a homogeneous raw material for printing, followed by the subsequent pyrolysis process. The cured structure was subsequently pyrolyzed up to 1200 °C in an argon atmosphere to obtain a Si-O-C/SiC ceramic. The presence of a SiC filler decreased the amount of shrinkage. Infiltrating molten Si into the structure resulted in a higher density and improved the bending strength significantly to 220 MPa. Due to low linear shrinkage of 3% after pyrolysis, they proposed the SLC approach for near-net-shape forming of ceramics.

### 2.3. Laminated Object Manufacturing (LOM)

In this method, thin sheets of material bond together to form 3D objects. A stack of sheets can be first laminated and then cut (cut-off-the-stack) or each sheet can be cut and then laminated (cut-then-bond) together [117]. The cutting is computer-controlled and follows the contour of the object’s shape in each sheet and removes the excess part to achieve the desired shape. In the cut-then-bond method, a layer of adhesive is applied to bind the sheets together. After that, heat and pressure are applied using heated plates or rollers to ensure firm fusing of the sheets. The sequence of the cutting to binding step is repeated until the full 3D object is built. Trimming with mechanical or laser cutting, depending on the material and desired specifications, may be required. LOM is commonly used for simple geometries and large-scale object fabrication in various industries.

Sieber et al. in 2000 achieved SiOC ceramics by infiltrating cleaning papers with a PCP-based slurry [118]. The slurry includes polymethylsiloxane (NH21), Si powder as an active filler, and α-SiC as a passive filler. Dried sheets after infiltration were laminated at 230 °C by hot pressing for 20 min followed by pyrolysis in an argon atmosphere at 1450 °C. Converted cellulose fiber to carbon fibers reacted with Si powder in the slurry and produced a SiC phase. By modifying the filler content in the slurry, laminated composites with low shrinkage were successfully demonstrated. Windsheimer in 2007 fabricated Si-SiC Composites with the LOM method [42]. Cellulosic PCP sheets were made using 76.8 wt% SiC powder, 20 wt% cellulose pulp, and a 3.2 wt% retention agent and binder. To bind the sheets, a polysiloxane-based adhesive coating was sprinkled on the surface followed by heating at 90 °C to distribute the coating homogenously. Laminated Si-SiOC composites were first sintered at 800 °C under a nitrogen atmosphere followed by infiltration of molten Si at 1500 °C to reduce the porosity. Fabrication of the highly dense composites is influenced by the orientation of the layers and loading directions, varying the bending strength to range between 150 MPa and 315 MPa. Based on the reliable and reproducible results achieved by Windsheimer, it may be possible to substitute commonly used binders for the fabrication of laminated composites with PCPs.

### 2.4. Extrusion-Based AM

As mentioned earlier, PCPs exhibit thermoplastic behavior because of their polymeric nature. As a result, extrusion-based additive manufacturing techniques can be utilized to create PDC parts [119]; however, they behave differently compared to the typical engineering polymers due to their low glass transition temperature and lack of chain entanglements [5]. Additionally, temperature significantly influences their viscoelastic properties [120]. To preserve the printed shape during the pyrolysis step, it is important to crosslink the PCP. It is worth noting that the introduction of fillers to PCPs can improve the shape stability regardless of crosslinking, as the fillers themselves can provide a higher viscosity and a network that will result in a yield point to retain the form [5].

#### 2.4.1. Direct Ink Writing (DIW)

Direct ink writing (DIW), also called robocasting [121], involves a controlled deposition of inks and pastes to create 3D objects [122]. The ink or paste is typically a mixture of polymers and fillers such as ceramics, metals, or composites with a careful formulation to achieve the desired rheological properties. In this process, a deposition system controls the flow and pressure of the ink extruded from the nozzle. The extruded material flows as a stream or filament and is deposited on a build platform. Movements of the extrusion system in multiple axes are controlled through a computer and follow a specific path to achieve the desired shape. The ink should have the necessary flow characteristics, including a proper yield stress and storage modulus to be extruded through a printing nozzle. An ideal ink is extruded by applying a high shear rate and at low viscosity [123], exhibiting a shear-thinning behavior. After deposition on the printing bed, the viscosity yield point should be immediately achieved by building a network structure to reduce deformation of the printed structure. As a result, a time-dependent oscillating viscosity analysis is of great importance.

To achieve a suitable rheology, the following methods are outlined below:

- High solid loading of the ink/paste formulation: Using a high solid content, forming a network of the extruded material happens fast [124]. However, only nozzles with a diameter of approximately 500 μm are applicable to avoid clogging of the nozzle.
- Addition of polymeric binder: Organic binders such as polyvinyl butyral (PVB) or polyethylene glycol (PEG) can be added to the ceramic phase (maximum of 23 wt%) [125]. In this way, the rheology of the ink can be justified without manipulating parameters such as pH.
- Reversible gel transformation: In this approach, ink is extruded in a non-wetting bath, often oil [126]. To achieve a reversible gelling effect, ceramic suspension is flocculated in a controlled manner by introducing polyelectrolytes, manipulating pH or ionic strength of the solvent. Addition of a gelling aid, like inverse thermoreversible gels, can be used alternatively.
- Use of preceramic polymers: Polysiloxanes and polysilsesquioxanes can be incorporated to control the rheology of the ink PCPs and offer a dual role [123]. The PCPs have the potential to serve as reactive binder additives, since they result in SiO_2_ and SiOC ceramics after pyrolysis in air or an inert atmosphere, respectively. When active fillers are added to the yielded SiO_2_, various silicate ceramics can be produced.

Depending on the nature of the ink or paste, a post-processing step such as curing or drying is required to achieve proper mechanical properties. The DIW method is a common option for applications where surface quality is not of the highest priority. A summary of the existing research on the direct ink writing (DIW) technique of polysiloxane and polysilsesquioxane PCPs can be found in Table 4.

Wei et al. in 2019 used UV-assisted DIW to fabricate SiOC cellular structures using a mixture of commercial polymethylsilsesquioxane (MK) and 3-(trimethoxysilyl)propyl methacrylate (TMSPM) [127]. After the synthesis of UV-curable MK-TMSPM with sol-gel reactions, trimethylolpropane triacrylate (TMPTA) was added as a low-viscosity curing agent to enhance the curing and achieve a proper viscosity. Photoinitiators of 1-hydroxy cyclohexyl phenylketone and ethyl phenyl(2,4,6-trimethylbenzoyl) phosphinate were also used to increase the absorption range of the UV light. The prepared mixture was printed with a filament size of 200 μm. In order to attain simultaneous and uniform curing, the laser beam is split into two beams with equal intensity and positioned on either side of the printing needle. Direct crosslinking of the material during printing prevented the formation of cracks during the pyrolysis process and resulted in only linear shrinkage of 25% and a mass loss of 30%.

By adding active fillers, various silicate compositions in the form of scaffolds were printed using polymethylsilsesquioxane (MK) as polysilsesquioxane. Zocca et al. in 2016 used MK mixed with ZnO and CaCO_3_ to produce hardystonite (Ca_2_ZnSi_2_O_7_) bioceramic scaffolds [123]. After ball milling the components in an isopropanol medium, the obtained homogenous ink was deposited in a non-wetting oil bath using a nozzle with a diameter of 0.41 mm, and scaffolds with dimensions of 15 mm × 5 mm × 5 mm were fabricated. In another study, Fiocco et al. in 2017 fabricated silica-bonded calcite scaffolds with the DIW technique [128]. MK was mixed with fused silica and CaCO_3_. The same mixing and printing parameters were used to achieve scaffolds. Elsayed et al. in 2019 used a mixture of methyl-siloxane (MK) and methyl-phenyl-siloxane (H62C) as silica sources to produce Biosilicate^®^ scaffolds [65]. A nozzle with a diameter of 0.81 mm was used for printing. More studies on akermanite (Ca_2_MgSi_2_O_7_)-based 3D scaffolds were fabricated by Dasan et al. in 2019 [129]. Three-dimensional scaffolds were fabricated using DIW with the synthesis of a bioactive glass–ceramic from a mixture of MK and a CaO-Na_2_O-B_2_O_3_-SiO_2_ glass system [130]. Complex hardystonite glass–ceramic scaffolds were obtained with DIW [85]. The amorphous phase obtained with SIRLES MK exhibited better reactivity with other additives compared to conventional silica sources. In most of the mentioned studies, fused silica was added to the ink as a thixotropic agent to adjust the viscosity for the DIW process. To achieve a hierarchical SiOC porous structure, MK and polymethyl methacrylate (PMMA) sacrificial microbeads with a diameter of 0.46 μm were used [131].

#### 2.4.2. Fused Deposition Modeling (FDM)

**Figure 10:**
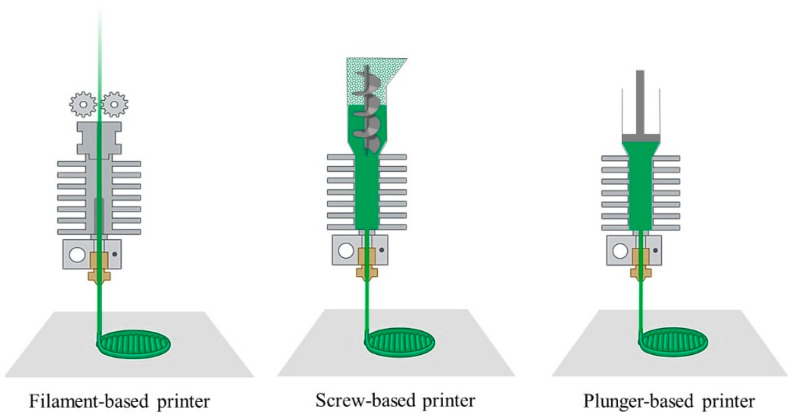
Different possibilities for extrusion-based additive manufacturing (created with BioRender.com).

Fused deposition modeling (FDM), also called fused filament fabrication (FFF), is based on extruding and deposition of thermoplastic materials [132,133]. FDM is one of the most widely used and accessible methods of 3D printing. In this method, thermoplastic feedstocks containing an organic binder and ceramic powder in the form of filaments or pellets are fed into an extrusion chamber inside a heat zone where the feedstock is melted (see Figure 10) [134,135]. The melted filament/pellets are pushed further and extruded through a small nozzle on a build platform layer by layer. The movements of the nozzle along the X and Y axes follow the pre-determined path of the object’s digital model. Each layer solidifies directly after deposition and bonds with the previous layer to ensure the structural integrity of the object. This layer-by-layer approach continues until the entire object is formed. After that, the printed object requires a debinding step to remove the organic thermoplastic component.

FDM printing of PDCs remains an underexplored area because the commonly used filament printers require spooling of the feedstock into filaments. Considering the high glass transition temperature (>50 °C) and melting temperature (ranging approximately from 70 °C to 250 °C) of PCPs, their filaments can be brittle and hard to spool. On the other hand, the thermoplastic nature of PCPs is favorable for adjusting the viscosity and elastic properties of filaments. To address this topic, Gorjan et al. [38] used a mixture of polymethylsiloxane (MK) and ethylene vinyl acetate (EVA) as thermoplastic components to produce PCP-based filaments for the FDM process. A mullite honeycomb structure and cylindric porous scaffolds were obtained by employing MK as a silica source and γ-Al_2_O_3_ as an active filler. After printing the structure using a nozzle with a 1.0 mm diameter at 170 °C, heat treatment with a gradual heating program was performed up to 1000 °C in air to crosslink and pyrolyze the preceramic on one hand and eliminate the organic components on the other hand. Further sintering up to 1550 °C in air was required to form mullite ceramic components through the reaction of alumina and polymer-derived silica. The resulting ceramic components exhibited no shape distortions or cracks. This work represented PCPs as viable candidates for filament preparation. Nevertheless, further research is necessary to obtain defect-free PDCs [18,38].

Gorjan et al. in 2019 used two different alumina sources with a D_50_ of 5.3 μm and 14.8 μm to produce mullite ceramics [38]. Complete mullitization was achieved at 1550 °C, as the reaction pathway necessitated higher temperatures in comparison to a protocol in which nano-sized alumina particles were employed [37]. Using alumina with a D50 of 5.3 μm, a residual corundum peak was observed even at 1550 °C. The use of a solvent to mix the components, specifically dissolving the PCP, and subsequent addition of fillers appear to be a more efficient approach for achieving a uniform dispersion and higher mullitization [37].

**Figure 11:**
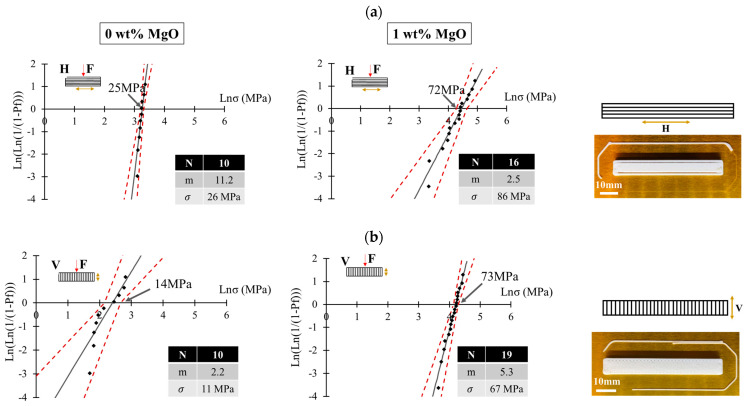
Weibull distribution of test specimens with (**a**) horizontal and (**b**) vertical printing orientations using 0 and 1 wt% MgO (reproduced from [136]). Open access CC-BY.

**Figure 12:**
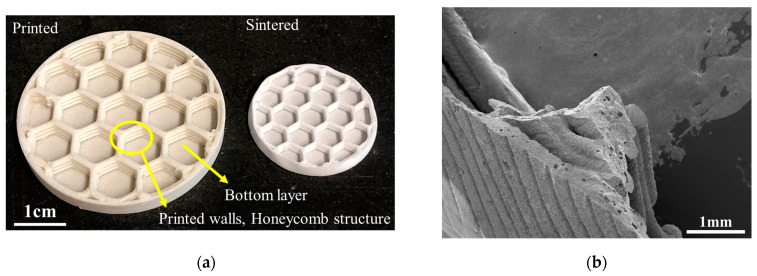
(**a**) Printed and sintered lightweight honeycomb substrate, (**b**) cross-section of the sintered honeycomb substrate [136]. Open access CC-BY.

Sarraf et al. in 2021 investigated the role of MgO as a sintering additive to enhance the sinterability and, subsequently, mechanical properties of mullite-based PDCs [136]. Employing 1 wt% MgO and a 5 h dwell time at 1600 °C, dense pure mullite structures were successfully produced. Only 0.5 wt% MgO was sufficient to avoid corundum impurity in the final mullite ceramic. The influence of printing direction on post-sintering mechanical strength, comparing ceramic structures with a 0 and 1 wt% MgO additive in both vertical and horizontal orientations, was studied as well. The addition of MgO was found to minimize the impact of printing orientation on flexural strength (Figure 11). The presence of closed porosity, appearing due to crosslinking reactions in the preceramic polymer, contributed to the lower Weibull modulus. By decreasing the wall thickness of printed structures, fewer pores were detected as shorter diffusion paths were needed for the escape of gaseous byproducts (Figure 12).

Thermal processing under both air and inert atmospheres during the crosslinking and pyrolysis steps of a commercial methyl-silsesquioxane preceramic polymer (MK) has been investigated by Sarraf et al. [137]. They reported that employing low heating rates (below 2 K/min) under an air atmosphere has a notable impact on the SiO_2_ yield of MK. Using a heating rate of 0.3 K/min and 0.6 K/min resulted in SiO_2_ yields of 69.1 wt% and 75 wt%, respectively, in contrast to the standard yield of 82 wt%. They concluded that slower heating rates provided more time to evaporate volatile species before the crosslinking occurred. In the FDM process, low heating rates and dwell times are necessary to remove binders without causing defects like cracks, blisters, and bubbles. To overcome this issue, mixing of feedstocks above the crosslinking temperature of MK (190 °C) was suggested. In this way, a stable yield of 81 wt% SiO_2_ was achieved, regardless of the heating rate.

In another study conducted by Sarraf et al. in 2022, the challenge toward the fabrication of bulk polymer-derived ceramics due to intensive gas evolution during crosslinking was addressed [138]. Using a mixture of ethylene vinyl acetate (EVA) and polyvinyl alcohol (PVA) as the binder system was sufficient to introduce controlled open porosity through a solvent debinding step. In this way, interconnected channels are created before the crosslinking of the preceramic polymer and generated gases from the crosslinking reactions can be removed. A pellet extruder was employed for 3D printing, and a crucial 50 vol% PVA binder content was identified to facilitate successful solvent debinding in water. The study investigated the impact of PVA content and different EVA grades on printability and debinding behavior. The mixing and printing process was improved when EVA with a lower melt flow index (MFI) was used. Higher vinyl acetate content of EVA was advantageous for subsequent thermal debinding, having higher gas permeability. Therefore, using a combination of PVA and EVA with higher vinyl acetate content, the fabrication of large PDCs can be feasible.

Nevertheless, when dealing with large structures, the solvent and thermal debinding, particularly the thermal debinding of the polymeric binder and pyrolysis, require careful consideration due to the higher amount of released gases and greater volume shrinkage. The thermoplastic binder exhibits a gradual decrease in viscosity, predictable with the Arrhenius equation, until binder decomposition starts. A decrease in viscosity along with the effect of gravity forces can become a critical factor for structural stability and may lead to distortion. To avoid this issue, crosslinking of the material before shaping can promote the stability of the structure and may be a viable strategy. However, additional research is needed to confirm the efficacy of this approach.

## 3. Conclusions and Prospects

The introduction of the polymer-derived ceramic (PDC) route in 1975 opened up a new door to explore novel possibilities in ceramic synthesis. Although most of the available studies on the fabrication of PDCs are performed in an inert atmosphere to obtain non-oxide ceramics, the synthesis under air to produce several oxide ceramics has been reported as well. In this review paper, synthesis of SiOC and silicate ceramics using pyrolysis of polysiloxane and polysilsesquioxanes in an air atmosphere has been explored, with a focus on incorporating active fillers into the PCP matrix, highlighting formation of mullite, wollastonite, cordierite, forsterite, and akremanite ceramics.

The possibility to combine the shaping and synthesis steps is an attractive aspect of polymer-derived ceramics. Preceramic polymers can be dissolved in many organic solvents and also be processed with thermoplastic shaping methods due to their polymeric nature. As a result, there is a continuous and growing adoption of various additive manufacturing (AM) techniques for the production of PDC structures. Various studies have presented interesting findings regarding the additive manufacturing (AM) of PDCs, indicating a promising future for these materials. Several studies have investigated the fabrication of bioactive ceramics/glass ceramics, SiOC, and mullite-based foams and scaffolds using vat polymerization and direct ink writing techniques. Also, the fabrication of mullite honeycomb structures has been reported via the fused deposition modeling technique. Vat polymerization and direct ink writing have been only used for the fabrication of thin and detailed PDC structures, foams, and scaffolds. Using the fused deposition modeling technique, however, there are first reports on the fabrication of bulk PDCs by modifying the binder system. There is a great potential in development of bulk and large PDC structures with high temperature stability and shock and creep resistance by using this method.

We expect that the constant improvement in hardware and software will further enhance the resolution capabilities of different AM techniques, enabling the production of even finer and more precise, detailed structures. Due to the development of multi-head printers, the ability to print with multiple materials in a single process is likely to facilitate the creation of hybrid PDC structures.

## Figures and Tables

**Table 1:** Various organosilicon polymers are used for the fabrication of PDCs (synthesis methods, obtained ceramics, and their applications) [5]. Open access CC-BY 4.0.

Organosilicon Polymer	Backbone Structure	Synthesis Methods	Applications
Polysilane	-R_1_R_2_Si-	Wurtz-type coupling of halosilanes, anionic polymerization of masked disilenes, catalytic dehydrogenation of silanes, reduction of dichlorosilanes	Photoresists, photo conductors, semiconductors, and precursors for synthesis of polycarbosilane
Polycarbosilane	-R_1_R_2_Si-C-	Kumada rearrangement of polysilane, ring opening polymerization, dehydrocoupling reaction of trimethylsilane, hydrosilylation of vinylhydridosilanes, grignard coupling reaction of (chloromethyl)- triethoxysilane and vinylmagnesium bromide	Precursors for preparation of SiC, electric- or photoconductors, photoresist nonlinear optical materials
Polysilazane	-R_1_R_2_Si-N=	Ammonolysis reactions of chlorosilanes with ammonia or with aminolysis, ring opening polymerization of cyclic polysilanane	Precursors for preparation of Si_3_N_4_ or SiCN, barrier for heat exchanger or on steel against oxidation
Polysiloxane	-R_1_R_2_Si-O-	Ring-open polymerization of cyclic silaethers, polycondensation of linear silanes	Precursors for preparation of SiOC, medicine electronics, textile chemistry
Polysilylcarbodiimides	-R_1_R_2_Si-N=C=N-	Pyridine-catalyzed polycondensation reaction of chlorosilanes with bis(trimethylsilylcarbodiimide)	Precursors for preparation of SiCN
Polyborosilazane	-R_1_R_2_Si-N(R_3_R_4_B)-	Co-condensation reaction of boron trichloride, organodichlorosilanes, and hexamethyldisilazane	Precursors for preparation of SiCBN

**Table 2:** Fabricated silicate and oxynitride ceramics from preceramic polymers and fillers.

Silicate Ceramic	Preceramic Polymer	Active Filler	Additive	Ref.
Mullite (3Al_2_O_3_·2SiO_2_)	MK	γ-Al_2_O_3_	-	[1,37,38,43]
	YR3370		-	
	MK+H62C		-	
	H62C		Borax	
ZTM (Zirconia Toughened Mullite)	MK	γ-Al_2_O_3_	ZrO_2_ and TiO_2_	[48]
Wollastonite (CaO·SiO_2_)		-	Ca-acetate	[49,50,51]
	MK	CaO	-	
		CaCO_3_	Hap	
	Mk+H62C	CaCO_3_	TEOS	
Yttrium mono-silicate (Y_2_O_3_·SiO_2_) Yttrium di-silicate (Y_2_O_3_·2SiO_2_)	MK	Y_2_O_3_	Eu_2_O_3_	[1,52]
		-	-	
		-	-	
Zircon (ZrO_2_·SiO_2_)	MK, H62C	ZrO_2_	TiO_2 _ Zircon seeds	[53]
Forsterite (2MgO·SiO_2_)	MK, H62C	MgO	TiO_2_	[54]
Willemite (2ZnO·SiO_2_)	MK	ZnO	Mn-acetate	[1]
Cordierite (2MgO·2Al_2_O_3_·5SiO_2_)	MK, H62C	γ-Al_2_O_3_, MgO	-	[55]
Gehlenite (2CaO·Al_2_O_3_·SiO_2_)	MK	γ-Al_2_O_3_, CaCO_3_	Eu_2_O_3_, CeO_2_	[56]
Akermanite	MK, H62C	MgO, CaCO_3_	m-Hap, Borax	[57]
Hardystonite (2CaO·ZnO·2SiO_2_)	MK	γ-Al_2_O_3_, ZnO	Eu_2_O_3_	[1]
β′-SiAlON	MK, H44	γ-Al_2_O_3_	Si_3_N_4_, AlN, SiC	[58,59,60]
Y-Si-O-Ns	MK	Y_2_O_3_	Eu_2_O_3_, CeO_2_	[25]
Wollastonite-based silicate bioceramic	MK	CaCO_3_	AP40 glass (apatite–wollastonite system)	[61]
Wollastonite- and hardystonite-based ceramics	MK	ZnO, CaCO_3_	AP40 glass (apatite–wollastonite system)	[62]
Wollastonite–diopside foam	H62C, MK	Mg(OH)_2_, CaCO_3_, Na_2_ HPO_4_·7H_2_O	Ca/Mg-rich silicate glass	[63]
Lithium orthosilicate (Li_4_SiO_4_)	PMS	Lithium carbonate (Li_2_CO_3_)	-	[64]
Biosilicate glass–ceramic	MK+H62C	CaCO_3_, Na_2_CO_3_, and anhydrous sodium phosphate	Biosilicate^®^ glass frit powder	[65]

**Table 3:** Additive manufacturing methods for fabrication of polymer-derived ceramics (reproduced from [2,95] with permission from Elsevier).

AM Technique	Features	Feedstock Form	Forming Method	Printing Requirements	Resolution
Direct ink writing (DIW)	Easy operation, low cost, wide choice of materials, highly accurate and complex 3D architectures	Slurry	Extrusion	Appropriate viscosity and elastic properties	Few hundred micrometers to mm
Fused deposition modelling	Low operating cost, high speed and large size capability, reuse waste, low printing precision, limited extrusion temperature range	Filament	Extrusion	In filament/pellet state	mm
Stereolithography (SL)/ Digital light Processing (DLP)	Moderate cost, high efficiency, good surface quality and ease of processability	Slurry	Polymerization	Dissolvable and possesses sufficient photocurable moieties	
Two-photon polymerization (TPP)	High surface quality, high printing precision, low speed	Slurry	Non-linear polymerization	Dissolvable, crosslinkable with two-photon absorption moieties	sub μm
Selective laser sintering (SLS)	Complex 3D structures, slow speed, low shrinkage, low curing temperature, high dimensional accuracy	Powder	Powder fusion	Meltable with laser and curable via reactive groups	μm to mm
Binder jetting	Complex 3D structures, limited strength, and rough surfaces	Powder and slurry	Binder bonding	Dissolvable in solvents and act as binders	μm to mm
Inkjet printing	High printing resolution, low material waste, limited by printable inks, low printing speed	Slurry	Binder bonding	Low viscosity, rapid crosslinking, and high ceramic yield	Few hundred micrometers to mm
Laminated object manufacturing	Large-scale production, no complicated chemical/physical processes, low speed, low precision, high anisotropy	Sheet	Binder bonding and laser cutting	In sheet state	mm

**Table 4:** Summary of printed polysiloxane/polysilsesquioxanes using DIW technique.

PCPs	Fillers	Obtained Ceramic	Print Resolution (μm)	Porosity (vol%)	Compressive Strength (MPa)
MK	ZnO and CaCO_3_	hardystonite (Ca_2_ZnSi_2_O_7_)	300–500	Up to 80%	0.6 ± 0.2
MK	CaCO_3_	silica-bonded calcite	450	56–64%	2.9–5.5
MK	Active: CaCO_3_, Na_2_CO_3_, and anhydrous sodium phosphate Passive: Biosilicate^®^ glass frit powder (<5 μm)	Biosilicate glass–ceramic	600	60%	Average of 6.7
MK	CaCO_3_, MgO	Akermanite (Ca_2_MgSi_2_O_7_)	-	72.4 ± 2.9%	3.3 ± 0.6
MK and H62C	Active: ZnO, CaCO_3_, SrCO_3_, Mg(OH)_2_ Passive: Glass powder	Sr/Mg-doped hardystonite	840	73 ± 1	2.3 ± 0.7
MK	PMMA sacrificial microbeads	SiOC	400	74.9 ± 3.2	8.19 ± 3.06

## Data Availability

Not applicable.
